# Exploring AI-Driven Machine Learning Approaches for Optimal Classification of Peri-Implantitis Based on Oral Microbiome Data: A Feasibility Study

**DOI:** 10.3390/diagnostics15040425

**Published:** 2025-02-10

**Authors:** Ricardo Jorge Pais, João Botelho, Vanessa Machado, Gil Alcoforado, José João Mendes, Ricardo Alves, Lucinda J. Bessa

**Affiliations:** 1 Egas Moniz Center for Interdisciplinary Research (CiiEM), Egas Moniz School of Health & Science, 2829-511 Caparica, Almada, Portugal; jbotelho@egasmoniz.edu.pt (J.B.); vmachado@egasmoniz.edu.pt (V.M.); galcoforado@egasmoniz.edu.pt (G.A.); jmendes@egasmoniz.edu.pt (J.J.M.); ralves@egasmoniz.edu.pt (R.A.); 2 Bioenhancer Systems Ltd., Stockport SK3 0GF, UK

**Keywords:** peri-implantitis, machine learning, metagenomic data, dental implants, biomarkers, saliva, peri-implant biofilms

## Abstract

**Background:** Machine learning (ML) techniques have been recently proposed as a solution for aiding in the prevention and diagnosis of microbiome-related diseases. Here, we applied auto-ML approaches on real-case metagenomic datasets from saliva and subgingival peri-implant biofilm microbiomes to explore a wide range of ML algorithms to benchmark best-performing algorithms for predicting peri-implantitis (PI). **Methods:** A total of 100 metagenomes from the NCBI SRA database (PRJNA1163384) were used in this study to construct biofilm and saliva metagenomes datasets. Two AI-driven auto-ML approaches were used on constructed datasets to generate 100 ML-based models for the prediction of PI. These were compared with statistically significant single-microorganism-based models. **Results:** Several ML algorithms were pinpointed as suitable bespoke predictive approaches to apply to metagenomic data, outperforming the single-microorganism-based classification. Auto-ML approaches rendered high-performing models with Receiver Operating Characteristic–Area Under the Curve, sensitivities and specificities between 80% and 100%. Among these, classifiers based on ML-driven scoring of combinations of 2–4 microorganisms presented top-ranked performances and can be suitable for clinical application. Moreover, models generated based on the saliva microbiome showed higher predictive performance than those from the biofilm microbiome. **Conclusions:** This feasibility study bridges complex AI research with practical dental applications by benchmarking ML algorithms and exploring oral microbiomes as foundations for developing intuitive, cost-effective, and clinically relevant diagnostic platforms.

## 1. Introduction

Dental implants are an increasingly utilized alternative for replacing missing teeth, with an annual growth rate of 14–23% per year [1]. Peri-implantitis (PI) is a common inflammatory and biofilm-related disease that affects the surrounding tissues of the dental implant [2]. This occurs when bacteria biofilms grow around the implant, causing soft tissue inflammation and bone resorption, leading to the eventual removal of the implant [3]. Current prevention strategies are based on the analysis of risk factors such as hygiene and smoking habits to assess patient eligibility to undergo a dental implant procedure [4,5]. Unfortunately, identifying risk factors alone is insufficient for accurately predicting peri-implantitis, which exhibits an incidence between 15 and 20% [6,7]. As a result, there is a pressing need for effective predictive tools to assist clinicians in preventing and managing peri-implantitis to prevent dental implant failures [8,9].

The oral microbiome is composed of a diverse and complex community of hundreds of different species of microorganisms that inhabit the human mouth [10,11]. These include a wide range of bacteria, fungi, viruses, archaea, and protozoa, varying in diversity and abundance, depending on the status of oral health [12]. Several studies support the concept of characteristic microbiome profiling for PI, highlighting its potential as a diagnostic tool [13,14,15,16,17,18]. These propose a set of bacterial pathogens characteristic of PI subgingival biofilm, supporting the evidence of their association with the etiology of PI. However, the application of such bacteria as microbial biomarkers for predictive purposes before dental implant placement is inadequate, as they are found in increased abundance or frequency when PI is installed but not in the precedent phase. Saliva is a possible viable solution for predicting PI as it may have key species or combinations of species that play a role in PI development. Additionally, saliva holds potential as a non-invasive diagnostic tool for predicting PI as it may contain inflammatory markers, metalloproteinases, and key species or combinations of species involved in PI development [19].

Supervised machine learning (ML) is a computational technique that enables a computer algorithm to learn complex interactions from datasets with numerous variables and, consequently, the generation of a predictive model [20,21]. Depending on the algorithm, these models can perform binary phenotype classifications (yes/no), which, in turn, can be applied to disease diagnosis, prognosis and effective treatment assessment [20,22]. Support Vector Machines (SVMs), Random Forests (RFs) and Logistic Regression (LR) have been recently applied for the classification of PI based on patient clinical data, rendering model’s performances with sensitivities up to 66% and specificities up to 72% [9]. Although these performances are reasonably acceptable, they fall short of the ideal standards required for practical application in dental practices (>90%). Oral-microbiome-based classifiers for PI using an RF algorithm on plaque microbiome data have been attempted, rendering highly predictive power models [23]⁠. To date, saliva-microbiome-based classifiers have not been explored as a solution for PI prediction.

Developing bespoke machine learning (ML) algorithms and optimizing models is a complex and labor-intensive process, requiring the testing and refinement of hundreds of algorithms while considering combinations of methods, architectures, and customizations [21,22]. These can be further optimized for specific cases by tuning different hyperparameters and feature engineering techniques [21]. Auto-ML approaches are suitable solutions for effectively finding bespoke optimal ML models [21,24,25]. These are often based on genetic and/or evolutionary algorithms that explore and test the dataset accounting for various algorithms and hyperparameter combinations [21,24]. In this work, we apply auto-ML approaches to explore a wide range of ML algorithms for finding bespoke predictive models for PI using oral metagenomic data. Here, we further analyze the resulting models from saliva and subgingival biofilm to evaluate their potential as predictive tools for use in dental practices.

## 2. Materials and Methods

### 2.1. Oral Metagenomic Datasets Acquisition and Information

A total of 100 metagenomes from the NCBI SRA database under BioProject number PRJNA1163384 (https://www.ncbi.nlm.nih.gov/sra/?term=PRJNA1163384 (accessed on 1 November 2024)) were used in this study (see Table S1 in the Supplementary Materials). These data were obtained from 40 anonymized patients, with one or more dental implants. Of these, 20 had healthy implants (HI group), while the remaining 20 had co-occurrence of healthy and peri-implantitis-affected implants (PI group). Each individual provided a saliva sample and one or two subgingival biofilm samples. The data pertain to male and female patients over 18 years of age (excluding pregnant or breastfeeding women) seeking dental care at the Egas Moniz Dental Clinic, Almada, Portugal, between January 2023 and September 2023.

Metagenomic data processing involved quality filtering of sequenced reads using Trimmomatic version 0.39 [26] with the following parameters: (1) removal of sequencing adapters, (2) trimming of bases with an average quality score below Q25 within a 5-base sliding window, and (3) discarding reads shorter than 100 bases. High-quality reads were then aligned to the reference human genome sequence assembly GRCh38/hg38 using Bowtie version 2.5 [27] to exclude host-derived sequences. The remaining high-quality sequences from each sample were assembled de novo using metaSPAdes version 3.15.5 [28] with default settings. The relative abundances based on mapped read counts of each identified bacteria and archaea species were obtained using the MetaPhlAn version 4.0.6 [29] computational tool and using the MetaPhlAn clade-specific marker genes mpa_vOct22_CHOCOPhlAnSGB_202212 database. Viral and fungi species read counts were obtained using the Kraken2 version 2.1.1 software tool and using the 2019 viral genome and fungi genome Kraken databases [30]. Abundance estimation at the species level was computed with Bracken version 2.9 using a Bayesian re-estimation method with Kraken [31]. Abundances were further normalized to the sample total read counts and expressed in ppm. Comprising all collected microorganisms’ relative abundances (ppm), we constructed two datasets in csv files, one containing all subgingival biofilm samples (SF1) and another containing the data of all saliva samples (SF2). These datasets include all bacteria, fungi and viruses identified in the study samples, including those associated with healthy implants and with peri-implantitis (Table 1). Metadata files containing the association of each sample with taxa were constructed for the biofilm and saliva datasets.

### 2.2. Statistical Analysis

To assess the statistical significance of microorganism abundances between healthy and PI groups, we employed the non-parametric Mann–Whitney U test, which is suitable for comparing two independent groups when the data may not follow a normal distribution. The two-tail Mann–Whitney U test was systematically applied to compare the abundance of microorganisms. We implemented the statistical analysis in Python 3.8 using the SciPy library version 1.10. Specifically, we used the mannwhitneyu() function from the SciPy.stats module to perform the Mann–Whitney U tests. For each test, we computed the U statistic and the associated *p*-value, which indicates the likelihood that the observed differences between groups occurred by chance. A *p*-value threshold of 0.05 was used to determine statistical significance. Only microorganisms with *p*-values lower than this threshold were considered significant, representing a probability of less than 5% that the observed differences between groups were due to random variation.

### 2.3. Single-Microorganism Classifiers

Single-microorganism binary PI classifiers (yes/no) were defined based on an abundance cut-off values for microorganisms identified with statistical significance between HI and PI groups. For cases where the average microorganism abundance in the PI group was higher than in the HI group, the classifiers were set to predict PI if the abundance value exceeded the cut-off value; otherwise, the model predicted a healthy implant. Conversely, microorganism abundance in the PI group was lower than in the HI group, and the classifiers predicted PI if the abundance value fell below the cut-off value; otherwise, a healthy implant was predicted. Abundance cut-offs were computed based on finding the value within the maximum and minimum range, resulting in the optimal sensitivity of the classifier tested with the datasets described in Section 2.1. Classifiers and all required parameter estimations were implemented in Python 3.8.

### 2.4. ML-Based Microbiome Classifiers

The Tree-Based Pipeline Optimization Tool (TPOT) version 0.12.0 was used for the generation of binary classifiers (yes/no) of PI based on the entire microbiome data. TPOT is an open-source software Python package for the automated generation of ML-derived predictive tools [24,32]. TPOT is an auto-ML approach that relies on genetic programming to generate predictive models with optimal performance, testing a wide range of ML algorithms and hyperparameter tuning [24]. The Python TPOT Classifier method was used for model training, testing and optimization. The method was implemented in a Python script for the systematic generation of models as previously implemented for other systems [33]. We set TPOT Classifier to perform 100 optimization attempts (generations), which randomly selected 50 distinct ML algorithms (population size) with a 5-fold cross-validation setting for training and testing of generated models. Here, the optimal Receiver Operating Characteristic–Area Under the Curve (ROC-AUC) was set as the optimization criterion. Model generation scripts were run on a 16-core Virtual Private Server with a 2.6 GHz processor.

### 2.5. ML-Driven Microorganism Combination Scoring Classifiers

The O2Pmgen tool version 1.1 was used for the generation of binary classifiers (yes/no) of PI based on scoring combinations of microorganism abundances. O2Pmgen tool is an AI-driven auto-ML approach that relies on an evolution-inspired algorithm that finds the best combination of biomarkers under a multi-objective fitness function for selecting models with optimal sensitivity and specificity [25]. This tool is available online on the digital phenomics platform (https://digitalphenomics.com (accessed on 5 November 2024)). Using this approach, the AI algorithm conducts supervised ML on the uploaded data where the scoring of *n* biomarker combinations evolved by the AI using a proportion of the data for training always inferior to 50% of the dataset, leaving the remaining data for testing purposes [34,35]. In this approach, the AI was programmed to learn to combine alterations in data that reflected up-regulations, down-regulations, and microorganism binary presences in the training set (healthy vs. PI implants). Classifiers were generated using the scoring function (*S*), where *Pi* is the absolute distance between the median level of microorganism *i* on the PI group and the sample microorganism abundance value; *Ni* is the absolute distance between the median level of microorganism *i* on the HI group and the sample value; *Wi* is the enrichment of microorganism *i* on the PI group; and *n* is the total number of microorganisms combined in the model.

(1) $$S=\sum_{i}^{n}\frac{100\;Wi\;\left(-Pi+Ni\right)}{Pi+Ni}.$$

### 2.6. Classifier Performance Analysis

Generated classifiers (predictive models) were evaluated by calculating performance metrics such as ROC-AUC, sensitivity, and specificity [36]. The ROC-AUC metric was calculated to assess the ability of the model to distinguish between positive and negative classes. The ROC curve data points were generated and the AUC was computed based on the area under this curve, which we calculated using a 5% interval window for higher precision [36]. Sensitivity (True Positive Rate) and specificity (True Negative Rate) were calculated to evaluate the model’s ability to correctly classify positive and negative instances, respectively [36]. These metrics were computed using standard formulas:

(2) $$Sensitivity=\frac{True\;Positives\;}{True\;Positives+False\;Negatives\;}$$

(3) $$Specificity=\frac{True\;Negatives\;}{True\;Negatives+False\;Positives\;}$$

Data visualizations were generated to provide insights into the model’s performance and the distribution of features. Boxplots, bar plots and pie charts were conducted using the matplotlib and seaborn libraries. For data manipulation, model performance filtering and ranking, we used the Pandas and numpy Python libraries (e.g., array manipulations, numerical calculations). All analysis and visualizations were implemented within a Jupyter Notebook using Python 3.8. Custom Python functions were written to automate the calculation of key performance metrics, including ROC-AUC, sensitivity, and specificity. Model evaluation code was designed to run iteratively, allowing for easy comparisons between different models and model configurations.

## 3. Results

**Figure 1:**
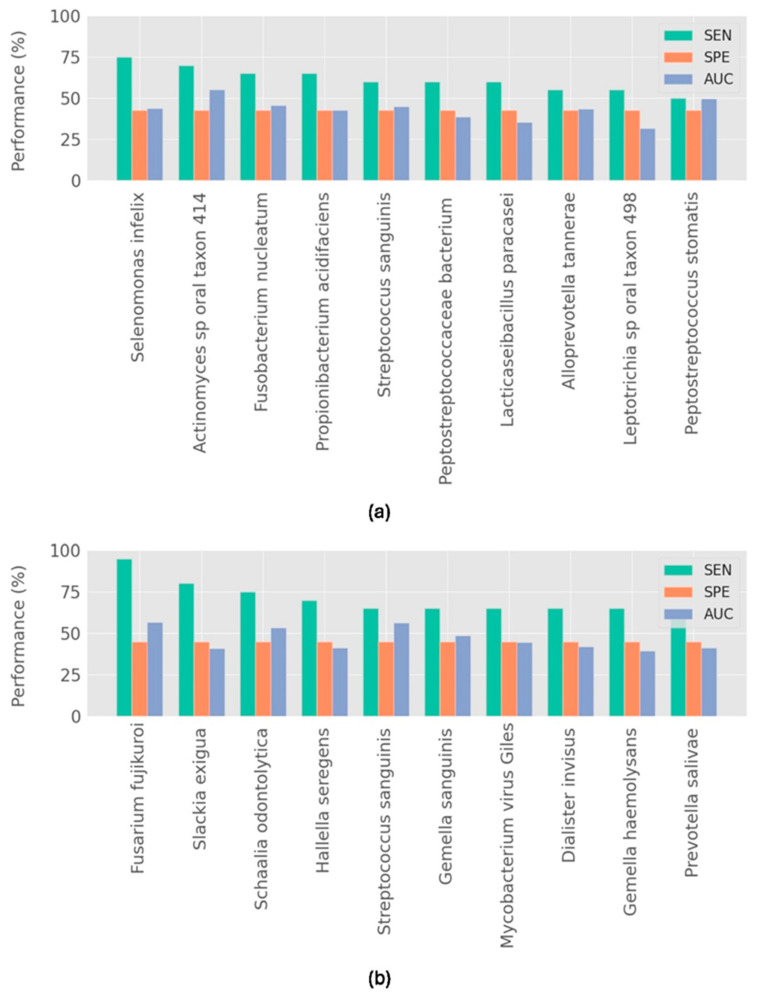
Top 10 best single biomarker predictors for (**a**) biofilm data, (**b**) saliva data. Performance metrics for each microorganism biomarker are depicted by different colors, where each color indicates the model’s sensitivity (SEN), specificity (SPE) and the ROC-AUC.

Statistical analysis of saliva and biofilm datasets identified 60 and 47 microorganism species, showing abundances significantly different between the HI and PI groups, respectively (see Table S2 in the Supplementary Materials). These results indicate putative biomarker candidates for PI classification based on their relative abundance in a peri-implant biofilm or saliva. Among the putative biomarker microorganisms identified, only *Stomatobaculum longum*, *Streptococcus sanguinis* and *Treponema denticola* were statistically significant in both biofilm and saliva microbiome. We generated PI classifiers based on a single microorganism with statistical significance from biofilm and saliva origins. We calculated the performance of each of these single biomarker PI classifiers and ranked them according to their predictive potential. The top 10 predictive biomarkers for biofilm data demonstrated sensitivities of up to 75% (e.g., *Selenomonas infelix*), but specificities remained low, not exceeding 43%, with AUC values reaching a maximum of 56% (Figure 1a). Regarding the saliva data, we obtained top-ranked biomarkers with sensitivities of up to 95% (*Fusarium fujikuroi*) with very low specificities (up to 42%) and AUC values of up to 55% (Figure 1b). These results suggest that individual microorganisms from biofilm and saliva exhibit poor predictive performance, despite demonstrating high sensitivity. The results further reveal that only *Streptococcus sanguinis* appears among the top-performing single biomarkers, suggesting distinct PI biomarkers for biofilm and saliva samples.

**Figure 2:**
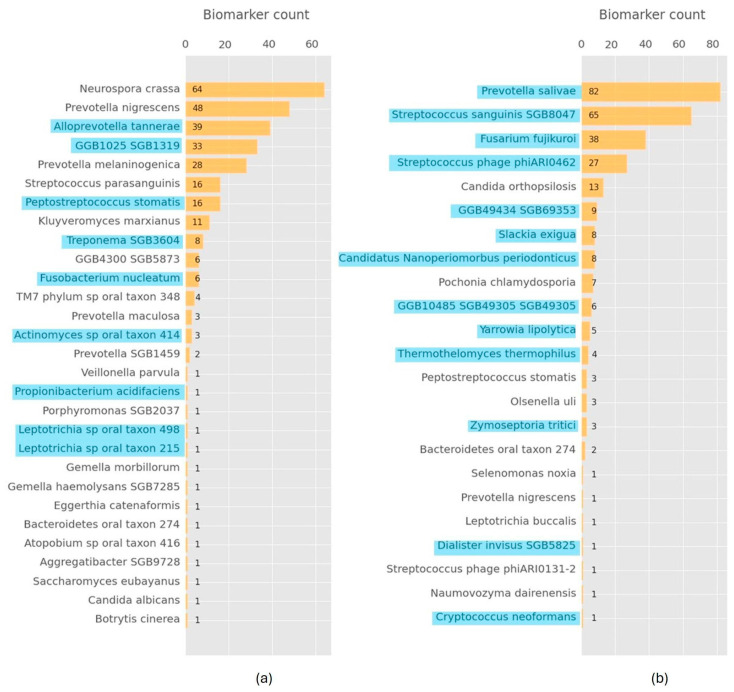
Frequency counts of the microorganisms found in the combinations of microorganisms found in the ML-driven biomarker scoring models: (**a**) biofilm microbiome data; (**b**) saliva microbiome data. Microorganism species names are depicted in the vertical axis and their counts in the horizontal axis. Names highlighted with a color indicate microorganisms identified with statistical significance.

AI-driven ML was applied to biofilm and saliva microbiome datasets to identify optimal combinations of microorganism species (biomarkers) that result in bespoke predictive tools for PI classification. We generated 100 models for each dataset based on the biomarker combinations scoring approach, rendering optimal predictive classifiers containing 2 to 4 combinations of different microorganisms. Using this approach, we obtained 35 distinct combinations of microorganisms for biofilm data PI classifiers and 23 distinct combinations for saliva data PI classifiers. These combinations are composed of 29 microorganisms for biofilm data (Figure 2a) and 23 for saliva data (Figure 2b). Interestingly, only around 30% of the identified biomarkers belonged to the microorganisms with statistical significance, suggesting a complex non-linear behavior. Our results further showed an over-representation of 3–5 biomarkers in the generated models, indicating consistent ML behavior that is incompatible with random selection. Specifically, *Neurospora crassa*, *Prevotella nigrescens* and *Alloprevotella tannerae* were prominent in the biofilm data PI classifiers, while *Prevotella salivae*, *Streptococcus sanguinis* and *Fusarium fujikuroi* were key biomarkers in the saliva data PI classifiers. Furthermore, our results showed very few common biomarkers between saliva and biofilm (*Prevotella nigrescens*, *Peptostreptococcus stomatis* and *Bacteroidetes* oral taxon 27).

**Figure 3:**
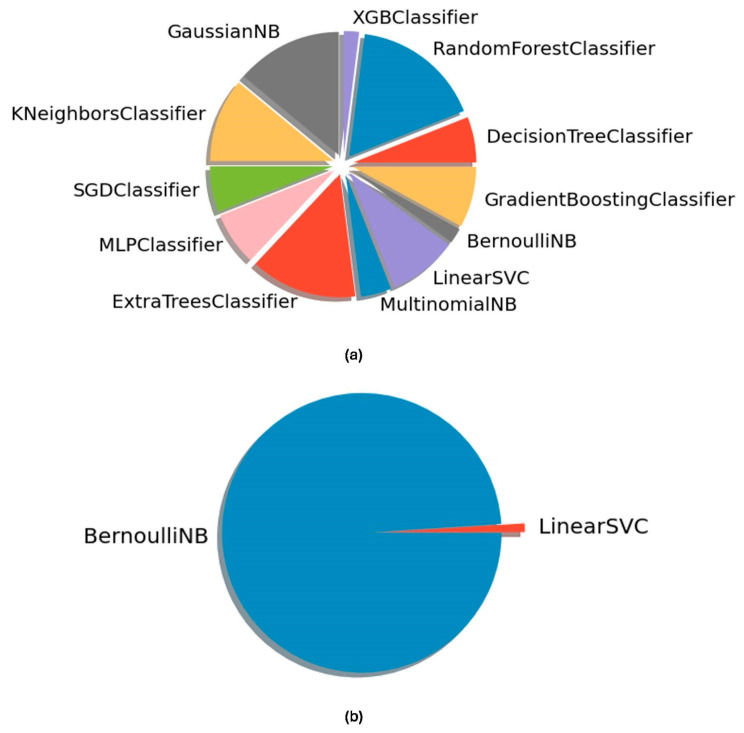
Optimal ML algorithms for the generated model classifiers using TPOT. (**a**) Algorithms obtained using the (**a**) biofilm dataset and the (**b**) saliva dataset. Names depicted in the pie charts indicate the name of the main algorithm behind the automatedly generated classifier pipelines, obtained from the TPOT framework. Variations in obtained pipelines, including pre-processing methods and hyperparameter settings, were not considered.

To explore the diversity of available ML algorithms that can be applied for predicting PI from microbiome data, we generated 100 models using an auto-ML framework. Using this approach, we tested 300 distinct AI-generated pipelines (model algorithms for binary classification) in each optimization attempt, resulting in 30,000 distinct model classifiers for each dataset. The obtained classifiers generated using biofilm data showed a variety of types of ML algorithms as optimal choices to be applied for these data (Figure 3a). Decision-tree-based classifiers such as the Random Forest and ExtraTrees classifier were the most frequent optimal solutions obtained in 39% of the AI-generated models. ML algorithms based on Bayes theorem such as Multinomial and Gaussian Naive Bayes classifiers were also present in 20% of optimal models generated. Less frequent (<10%) ML algorithms such as the artificial neural network MLP classifier were also obtained as optimal solutions. In contrast, classifiers generated using the saliva dataset showed only two types of optimal ML algorithms, the Bernoulli Naive Bayes (BernoulliNB) algorithm and the Linear Support Vector Classifier (LinearSVC) algorithm (Figure 3b). BernoulliNB was overrepresented in the generated models, accounting for 99% of the models, whereas LinearSVC appeared in only 1% of the generated models. These results highlight significant differences in the optimal ML algorithms for application to biofilm and saliva metagenomic data.

**Figure 4:**
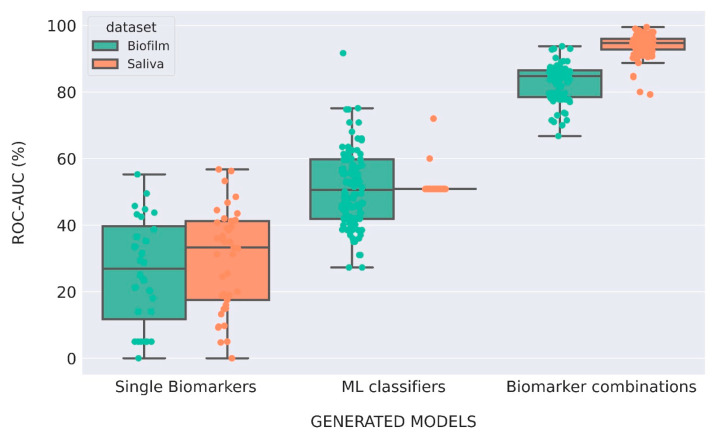
Comparison of the AUC values of the generated models using different approaches for biofilm and saliva data. The analysis of models from the biofilm dataset is depicted in green and the ones from saliva dataset depicted in orange. Boxplot limits indicate 95% intervals and the color-filled dots represent all AUC values’ distribution across the respective grouped dataset.

The ROC of the PI classifiers generated from biofilm and saliva microbiome data showed substantially higher AUC values for the ML-driven models when compared with the single-biomarker approach (Figure 4). Our results demonstrated that ML-driven models based on scoring combinations of biomarkers achieved AUC values ranging from over 70% to as high as 98%, indicating good to excellent predictive power. The results further showed that about half of the ML-driven models generated from saliva data exhibited higher AUC values compared to those derived from biofilm data. This positions ML-driven models based on scoring saliva biomarkers as having higher predictive power compared to those using biofilm data. Regarding the models based on ML algorithms, only 25% of these achieved AUC values above the acceptable threshold for reasonable predictive power (>60%), with a maximum of 90% for biofilm microbiome data and 65% for saliva microbiome data.

**Figure 5:**
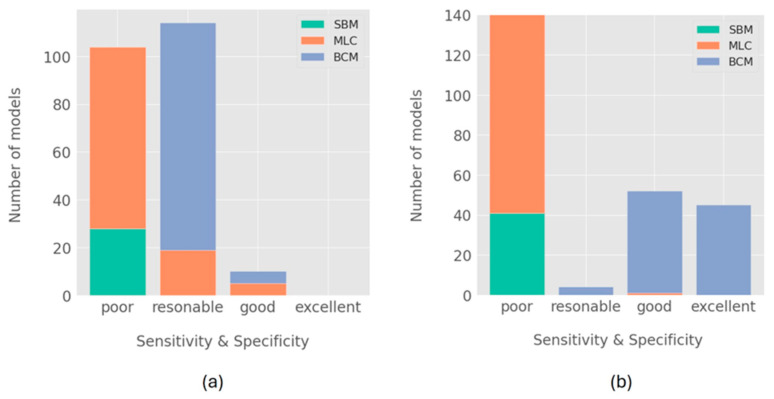
Number of generated models is classified according to distinct classes of performances: (**a**) models generated with biofilm dataset; (**b**) models generated with biofilm dataset. Single-biomarker models (SBM) are indicated in green, machine learning classifiers (MLCs) in orange, and biomarker combination models (BCMs) in blue. Classifiers with poor performance classification were considered to have a sensitivity or specificity below 70%. Reasonable-performance models were considered to have a sensitivity and specificity in the range of 70% up to 79%, whereas good performance was in the range of 80% up to 89%, and excellent models had a performance of above 89%.

The sensitivities and specificities of the generated PI classifiers revealed distinct performance categories across models and their respective explored approaches (Figure 5). Poor sensitivities and specificities for single-biomarker classifiers were obtained. For biofilm-derived PI classifiers, we identified 10 models with good performance, where sensitivities and specificities ranged from 80 to 90% (Figure 5a, see Table 2 for model details). Among these models, the 5 top-ranking performances were based on scoring combinations of 3–4 microorganisms with a predominance of *Alloprevotella tannerae*, *Prevotella nigrescens*, and *Neurospora crassa* (Table 2). The artificial neural network MLPClassifier applied to the entire biofilm microbiome data rendered a model on the 6th best ranking, with a sensitivity of 90% and a specificity of 85%. Similarly, Gaussian Naive Bayes and the Support Vector Machines SGDClassifier were also among the best-performing models. For the remaining ML-driven models, the majority of the models based on scoring biomarker combinations had reasonable performances (70–80%) in sensitivity and specificity. In contrast, the majority of the models that correspond to models based on ML algorithms applied to entire microbiome biofilm data often resulted in poor performances (sensitivity and specificity <70%), with only 19% of the models demonstrating reasonable performances.

For saliva-derived models, we identified 45 models with excellent performance (90–100%) in their sensitivity and specificity (Figure 5b). We also obtained 52 models with good performances (80–90%) in sensitivity and specificity. The majority of the models with good and excellent performances were ML-driven models based on scoring biomarker combinations. Only one model based on an ML algorithm had a good performance for saliva data. The top 10 best-performing PI classifiers for saliva data showed only ML-driven models based on the scoring of 2–4 microorganism species combinations (Table 3). Among these microorganism species, *Prevotella salivae* and *Streptococcus sanguinis* appeared in most models (8 and 7, respectively), including the top 3 best-performing models, which achieved 100% sensitivity and 95% specificity. Other microorganism species such as *Pochonia chlamydosporia* and *Fusarium fujikuroi* were also components of the top best-performing models, indicating alternative predictive combinations.

## 4. Discussion

Modern AI and ML frameworks are being progressively applied in dental and oral health research, including basic, translational and clinical research [37]. ML classifiers based on applying LR, SVM, and RF algorithms on demographic data and parameters known to be risk factors have been proposed as solutions for the prediction of PI [9]. Although the resulting ML classifiers showed acceptable predictive power (AUC 70–87%), these models have shown limited performance with reported sensitivities and specificities up to 66% and 72%, respectively [8,9].

In our study, we analyzed a wide range of ML algorithms to benchmark oral microbiome data as an alternative data-rich solution capable of resulting in predictive tools (classifiers) with performances suitable for its application to dental practices. From our results, the AI-driven ML classifiers based on scoring the abundances of 2–4 saliva microorganisms showed the highest performances and predictive power among all generated classifiers, including the ones derived from subgingival biofilm microbiomes and the single-microorganism approach. These model performances achieved remarkably high sensitivities (95–100%) and specificities (90–95%), suggesting that ML-driven scoring of microorganism abundances in saliva can be a suitable approach for the generation of alternative predictive tools that outcompete current approaches [8,37].

While the resulting models in this study demonstrate high performance and predictive power, there are several limitations that should be acknowledged. First, the sample size of 40 saliva and 60 biofilm samples, although sufficient for initial exploratory analysis, is relatively small. This limits the generalizability of the results and increases the risk of overfitting, particularly given the complexity of machine learning models, which may lead to over-optimistic performance metrics. Although advanced machine learning techniques were used, overfitting remains a concern, especially when the number of features (microbial taxa) exceeds the number of samples. Larger and more diverse datasets would be necessary to validate the robustness of the models and ensure their applicability to broader populations. Moreover, the study sample consists of exclusively Portuguese patients, which introduces population-specific biases. The microbiome varies across different populations, potentially limiting the application of the modes to other ethnic or geographic groups. Additionally, this study did not account for the analysis of demographic and clinical data, such as age, gender, smoking status, or systemic health conditions, which are factors that may influence microbial composition and disease outcomes. These unaccounted-for variables could act as confounding variables, potentially affecting the predictive accuracy of the models.

Another limitation arises from the use of metagenomic sequencing to profile bacteria, fungi, and viruses. While this approach enables a comprehensive analysis of the microbial community using AI and ML, it depends on reference databases for species identification, which may be incomplete or biased toward certain microorganisms. This database dependency introduces potential errors in taxonomic assignment, as novel or poorly characterized species may not be accurately identified, leading to misclassifications or omissions. Moreover, the bioinformatics pipelines used for abundance estimation can introduce additional sources of error, such as incorrect normalization or inaccurate taxonomic assignments due to database limitations. These factors could contribute to deviations in microbial abundance profiles and ultimately affect the performance and reliability of the predictive models.

Furthermore, this study did not explore the grouping of microorganisms based on functional or taxonomic characteristics, which could provide deeper insights into microbial interactions and their roles in peri-implantitis development. Grouping microorganisms into more meaningful clusters could enhance the predictive power of the models by focusing on functional pathways rather than individual taxa.

Therefore, although our study can already propose ML models for predicting PI with good performances above 80% of sensitivity and specificity, we can only draw qualitative benchmarking conclusions regarding optimal approaches and system design for future studies that aim to develop novel PI predictive tools. Despite the data limitations, our best-performing ML models from biofilm data rendered similar performances in comparison with the work of Ghensi et al. [23], who applied an RF algorithm to subgingival biofilm microbiome for the diagnosis of PI. This supports the idea that our models are consistent with work performed in a larger cohort of patients. Moreover, our work showed that ML algorithms such as the MLPClassifier, the Gaussian Naive Bayes and the SVM SGDClassifier may be alternative solutions for best-performing models using biofilm data.

The results from our study further support the idea that applying AI-driven solutions for finding and scoring bespoke microorganism combinations (biomarkers) for predicting PI is more effective than using a classical ML approach that uses algorithms such as LR, RF, SVM on the “entire” microbiome. This suggests a paradigm change that challenges the current reported models by using AI-driven approaches that perform the identification of optimal biomarker combination scoring models. This is a significant finding as models derived from a few combinations of biomarkers offer advantages for clinical applications. Those microbial biomarkers can potentially be identified using more affordable and straightforward tests, such as qPCR. In contrast, the sequencing costs for entire-microbiome determination and quantification using metagenomics techniques may constrain the application in dental practices of ML models that rely on the whole microbiome. Although these ML approaches account for more information, they are usually “black boxes” and prone to artefacts, bias and overfitting, which is difficult to identify [21,36,38]. On the other hand, models based on a few combinations of microorganisms are much less informative but offer the possibility of being assessed from the biological point of view.

Interestingly, our work showed few similarities between biofilm- and saliva-predictive biomarker species, identified in both single- and combined-biomarker classifiers. Further, their predictive potential in comparison with that of the best-performing models was reduced. This suggests that saliva and biofilm microbiomes are distinct ecosystems; however, saliva biomarkers are good proxies for PI prediction and biofilm biomarkers, showing high predictive power.

Using AI-driven ML applied to biofilm and saliva metagenomic datasets, we identified optimal combinations of microbial species that serve as bespoke predictive tools for PI classification. Notably, *Neurospora crassa*, *Prevotella nigrescens*, and *Alloprevotella tannerae* emerged as prominent biomarkers in the biofilm-based PI classifiers, while *Prevotella salivae*, *Streptococcus sanguinis*, and *Fusarium fujikuroi* stood out in the saliva-based PI classifiers.

*Neurospora crassa* and *Fusarium fujikuroi* are two fungal species. However, it is important to recognize that all microbial species within the oral cavity engage in inter- and intra-species interactions, which may play a crucial role in determining health or disease states [39]. *Neurospora* is generally considered non-pathogenic to humans, whereas *Fusarium* is recognized as an opportunistic pathogen; however, there is evidence that *Neurospora* can occasionally be implicated in infections, including those of the oral cavity and other body sites, highlighting its potential, albeit rare, role in human disease [40]. To the best of our knowledge, these fungi have not been identified as part of the PI microbiome.

*Prevotella nigrescens* and *Alloprevotella tannerae are* two common oral bacterial species, involved in oral inflammatory processes, the former being frequently found in dysbiotic biofilms of periodontal diseases and the latter in carious lesions [41]. These two species, among others, have also been found to be more abundant in the PI subgingival biofilm sites than in healthy implant sites [42]. Moreover, Verdugo et al. [43] reported that PI lesions were more likely to harbor *Prevotella nigrescens* than healthy implants, and saliva from patients with PI showed an even higher prevalence of *P. nigrescens*. Consistently, our findings identified *P. nigrescens* as one of the three biomarkers shared between saliva and biofilm datasets, alongside *Peptostreptococcus stomatis* and *Bacteroidetes* oral taxon 27. *Peptostreptococcus stomatis* has been reported to be more abundant in peri-implantitis than in periodontitis [14,44].

Studies with much larger datasets are still needed before such ML classifiers can be implemented into dental practices. In particular, datasets comprising several hundred saliva samples will enable validation using independent datasets that were not involved in model construction. Models that combine saliva microorganisms with demographic data and risk factors may also be an idea worth exploring in future work to develop best-performing PI models, applicable to dental practices. Finally, the development of affordable experimental testing platforms encompassing these ML models is still needed.

The implementation of advanced AI and ML strategies in dental practices presents significant challenges, particularly due to the complexity of interpreting such data by clinicians. Although contemporary AI frameworks have demonstrated high predictive accuracy, such as scoring microorganism abundances for PI prediction, these models often rely on intricate data structures that are not readily comprehensible to practitioners. Clinicians, who are primarily trained in diagnostics and treatment rather than computational analysis, may find difficulties in integrating these data-intensive systems into routine practice. Moreover, we should avoid leading AI-driven approaches to operate as "black boxes", rendering it challenging to translate their outcomes into actionable clinical decisions.

Researchers should simplify these technologies by converting complex ML outputs into intuitive classifiers. For instance, predictive tools could use smaller biomarker sets, such as specific microbial species combinations identified through affordable tests like qPCR, rather than relying on entire-microbiome metagenomic data. These tools would enhance accessibility while preserving high sensitivity and specificity, as recent studies have shown. These technologies can improve patient outcomes in dental settings by bridging the gap between complex AI research and user-friendly applications.

Acknowledging these challenges, our results show this new AI-based approach is feasible to predict peri-implantitis using microbiome data. By benchmarking ML algorithms and exploring saliva and biofilm microbiomes as predictive tools, this study lays the groundwork for developing intuitive, cost-effective, and clinically relevant diagnostic platforms.

## Figures and Tables

**Table 1:** Characteristics of metagenomic datasets. Subsets of microbiome data are grouped into two phenotypical dental implant outcomes, disease or health.

Characteristics	Biofilm Dataset	Saliva Dataset
Sample number	60	40
Number of patients	40 ^1^	40 ^1^
Total HI	40	20
Implants with PI	20	20
Healthy implants from patients with PI	20	0
Bacteria	596 ^1^	596 ^1^
Fungus	52 ^1^	52 ^1^
Virus	586 ^1^	586 ^1^

^1^ Same features in both datasets. HI: healthy implants, PI: peri-implantitis.

**Table 2:** Top 10 best-performing PI classifiers obtained from biofilm microorganism abundances. Models with sensitivities and specificities > 79%. were filtered. Classifiers were ranked based on a score that consists of the sum of the obtained sensitivity, specificity and AUC. Models are ranked in descending order by performance scoring.

Classifier Rank	Algorithm Type	Model Input Data (Abundances)	Sensitivity (%)	Specificity (%)	AUC (%)
1	Biomarker Scoring	*Alloprevotella tannerae* *Neurospora crassa* *Prevotella nigrescens*	95	80	89
2	Biomarker Scoring	*Prevotella nigrescens* *Prevotella melaninogenica* TM7 phylum sp oral taxon 348	90	80	88
3	Biomarker Scoring	*Alloprevotella tannerae* TM7 phylum sp oral taxon 348 *Botrytis cinerea* *Prevotella nigrescens*	90	83	83
4	Biomarker Scoring	*Alloprevotella tannerae* *Neurospora crassa* *Streptococcus parasanguinis*	90	80	85
5	Biomarker Scoring	*Alloprevotella tannerae* *Neurospora crassa* *Streptococcus parasanguinis*	90	80	84
6	MLPClassifier	Entire microbiome	90	85	75
7	GaussianNB	Entire microbiome	80	98	65
8	SGDClassifier	Entire microbiome	85	80	66
9	GaussianNB	Entire microbiome	80	85	62
10	GaussianNB	Entire microbiome	80	85	61

**Table 3:** Top 10 best-performing PI classifiers obtained from saliva microorganism abundances. Models with sensitivities and specificities > 94% were filtered. Classifiers were ranked based on a score that consists of the sum of the obtained sensitivity, specificity and AUC. Models are ranked in descending order by performance scoring.

Classifier Rank	Algorithm Type	Model Input Data (Abundances)	Sensitivity (%)	Specificity (%)	AUC (%)
1	Biomarker Scoring	*Prevotella salivae* *Streptococcus sanguinis* GGB10485 SGB49305 GGB49434 SGB69353	100	95	99
2	Biomarker Scoring	*Prevotella salivae* *Streptococcus sanguinis* *Pochonia chlamydosporia* GGB10485 SGB49305	100	95	98
3	Biomarker Scoring	*Prevotella salivae* *Streptococcus sanguinis* GGB10485 SGB49305	100	95	97
4	Biomarker Scoring	*Streptococcus* phage phiARI0462 *Fusarium fujikuroi*	95	95	97
5	Biomarker Scoring	*Prevotella salivae* *Streptococcus sanguinis* *Yarrowia lipolytica*	95	95	96
6	Biomarker Scoring	*Prevotella salivae* *Streptococcus sanguinis* *Pochonia chlamydosporia*	95	95	96
7	Biomarker Scoring	*Prevotella salivae* *Streptococcus* phage phiARI0462 *Fusarium fujikuroi*	95	95	96
8	Biomarker Scoring	*Streptococcus* phage phiARI0462 *Fusarium fujikuroi*	95	95	96
9	Biomarker Scoring	*Prevotella salivae* *Streptococcus sanguinis* *Pochonia chlamydosporia*	95	95	95
10	Biomarker Scoring	*Prevotella salivae* *Streptococcus sanguinis* *Pochonia chlamydosporia*	95	95	95

## Data Availability

The metagenomic data used are accessible in the NCBI SRA database (https://www.ncbi.nlm.nih.gov/sra (accessed on 1 November 2024)) under the NCBI BioProject ID PRJNA1163384.

## Supplementary Materials

The following supporting information can be downloaded at: https://www.mdpi.com/article/10.3390/diagnostics15040425/s1, Table S1: Information on the samples originating the 100 metagenomes deposited on NCBI SRA database under the BioProject number PRJNA1163384. Table S2: Statistical analysis results of saliva and biofilm datasets.
